# A Digital System to Assess Mental Health in People With Intellectual Disability (MENTALSED): Protocol for a Development and Pilot Study

**DOI:** 10.2196/106072

**Published:** 2026-09-30

**Authors:** Jairo Rodríguez-Medina, Clara Gonzalez Sanguino, Elena Betegón, Alba Ayuso-Lanchares, Teresa Boemo

**Affiliations:** 1 Department of Psychology, University of Valladolid, Paseo de Belén 1, Valladolid, Castille and León, 47011, Spain, 34 658010968; 2 Department of Social Psychology, Work Psychology and Individual Differences, Complutense University of Madrid, Madrid, Spain

**Keywords:** ecological momentary assessment, easy-to-read text, intellectual disability, objective measures, self-report, heart rate variability

## Abstract

**Background:**

People with intellectual disabilities have a high prevalence of mental health problems, which are often underdiagnosed and treated late due to difficulties in assessment. The limited availability of validated instruments, communication barriers, and reliance on external informants hinder the early detection of symptoms and the monitoring of their progression.

**Objective:**

This study describes the protocol for the development and piloting of MENTALSED, a digital assessment system designed for the continuous monitoring of mental health and emotional well-being in people with intellectual disability through ecological momentary assessment using traditional questionnaires supplemented with psychophysiological measures.

**Methods:**

The study uses a mixed methods design, combining quantitative and qualitative procedures. Following a literature review, a survey assessed the needs of direct care professionals. On the basis of these findings, self-report and proxy-report EMA questionnaires were developed. The professional version was evaluated by university experts and direct care professionals. The self-report version was adapted into an easy-to-read format with the support of a focus group of people with intellectual disabilities and subsequently evaluated by individuals with intellectual disabilities and professionals. The questionnaires were implemented on the Avicenna Research platform. Objective psychophysiological measures (heart rate variability via a chest strap and sleep quality and heart rate via a smartwatch) were included. All data are integrated into a custom application displaying 4 well-being dimensions. The subsequent pilot study will involve individuals with intellectual disability and support professionals over a 2-week period in natural settings.

**Results:**

The project was funded by the Ministry of Science, Innovation and Universities, the State Research Agency, and the European Regional Development Fund/European Union (grant PID2023-150190OA-I00/MENTAL-SED). The needs assessment and validation phases were conducted between January and May 2025. As of manuscript submission, a total of 91 participants (71 professionals and experts and 20 individuals with intellectual disability) have been recruited and have participated in the development and validation stages. The 2-week pilot study is currently concluding data collection. Data analysis for the pilot phase is expected to begin in October 2026, and the final results are expected to be published in 2027.

**Conclusions:**

MENTALSED is expected to facilitate the early detection of emotional and behavioral changes, improve diagnostic accuracy, and enable the development of evidence-based preventive interventions. The integration of multiple sources of information is expected to help overcome traditional limitations in mental health assessment in this population.

## Introduction

Mental health in individuals with intellectual disability constitutes an area of particular clinical, social, and socio-health relevance due to the high prevalence of comorbid psychopathology within this population. Among adults with intellectual disability, mental disorders are both common and more prevalent than in the general population, with estimates ranging from 10% to 39% [1], and a mean prevalence of 33.6% reported in more recent reviews, particularly among individuals with mild or moderate intellectual disability [2]. In the Spanish context, Peña-Salazar et al [3] found that 29.57% of participants presented a previously undiagnosed psychiatric disorder, highlighting both the high frequency of these conditions and the existing difficulties in their detection.

Regarding the types of psychopathology commonly associated with intellectual disability, Mazza et al [2] identified mood disorders (6.7%), anxiety disorders (5.5%), schizophrenia (4.8%), unspecified psychotic disorders (3.9%), and personality disorders (2.8%) as the most frequent diagnoses. Their findings also pointed to an increased vulnerability to psychotic spectrum disorders compared with the general population (approximately 1%). Similarly, Perera et al [4] observed that individuals with intellectual disability were between 9 and 10.8 times more likely to receive a diagnosis of a severe mental disorder and presented a slightly increased risk of depression. In addition, Deb et al [5] estimated the prevalence of severe functional psychiatric disorders to range between 14.4% and 22.4%, although this percentage may be even higher when depressive and anxiety symptoms, challenging behaviors, and other neurodevelopmental disorders are taken into account. Furthermore, Hsieh et al [6] reported prevalence rates of anxiety and depression of up to 12.3% and 15%, respectively, while in Spain, Peña-Salazar et al [3] found differences according to the severity of intellectual disabilities: major depressive disorder was more frequent among individuals with mild or moderate intellectual disability, whereas anxiety disorders were more prevalent among those with severe or profound intellectual disability.

Despite this high prevalence, the detection of mental health problems in individuals with intellectual disability remains limited. People with intellectual disability are at greater risk of underdiagnosis and of receiving insufficient care, which may be explained by difficulties in screening and detection, as well as barriers to accessing mental health services [3]. This issue may be related to the phenomenon known as diagnostic overshadowing, whereby certain emotional, cognitive, or behavioral symptoms are mistakenly attributed to the intellectual disability itself rather than interpreted as potential indicators of a comorbid mental disorder [7]. The assessment process is further influenced by the specific characteristics of the intellectual disability population. Cognitive and communication difficulties may negatively affect symptom expression and social interaction, thereby increasing emotional problems and complicating their identification [8,9]. In this regard, Karlsen et al [10] noted that recognizing mental health symptoms in adults with intellectual disability may be challenging for both professionals and families, particularly due to difficulties in obtaining self-reported information and the potential behavioral manifestation of certain emotional or psychiatric problems. Accordingly, European guidelines for the assessment and diagnosis of psychiatric disorders in adults with intellectual disability emphasize the importance of using clear and adapted language, conducting direct observations, monitoring variables such as activity level, sleep, and behavioral changes, using objective instruments, and promoting multidisciplinary assessments [5]. Consequently, there is a need to develop assessment systems that integrate accessible procedures, information from multiple sources, and objective measures to improve early detection and reduce the exclusive reliance on traditional instruments or external informants.

In this context, ecological momentary assessment (EMA) has been proposed as a promising strategy for improving mental health assessment processes, as it enables the repeated collection of real-time data in naturalistic settings [11]. This methodology facilitates the recording of thoughts, emotions, and behaviors in everyday life, as well as the analysis of the temporal dynamics of symptoms and their relationship with contextual factors. Moreover, advances in mobile technology have promoted the integration of assessments and interventions into daily life, expanding their application across different stages of the clinical process [12]. EMA enables the collection of information on recent or current experiences, thereby reducing biases associated with retrospective recall and improving the ecological validity of assessment [13]. It may also complement information obtained from external informants, whose reports can be affected by systematic biases or by the evaluator’s own emotional state [14]. In its electronic format, EMA allows behaviors to be recorded close to the time of occurrence and across multiple contexts, facilitating the detection of behavioral fluctuations and the characterization of behavior over time [11]. Nevertheless, although the available evidence suggests its feasibility in populations with cognitive impairment, lower completion rates have also been observed, underscoring the need to adapt protocols to the specific characteristics of these groups [13].

Across empirical studies, EMA protocols in intellectual disability show substantial heterogeneity in sampling frequency, duration, and item content. When EMA is applied to individuals with intellectual disability, methodological adaptations become critical to ensure feasibility. For example, Wilson et al [15] implemented a relatively intensive EMA protocol with 7 random notifications per day between 8 AM and 8 PM over 7 days. They used a mobile app adapted specifically for adults with mild to moderate intellectual disability, and the average compliance was only 33.8%. In contrast, lower-intensity EMA designs appear to be related to higher feasibility. Hulsmans et al [16] implemented a daily diary EMA protocol over 60 consecutive days with adolescents and young adults with mild intellectual disability or borderline intellectual functioning (n=50; mean IQ 72.6). Participants completed 1 assessment per day, typically scheduled at a convenient evening time (most commonly 8 PM). This lower-frequency design resulted in markedly higher feasibility, with an average compliance rate of 70.4%, increasing to 86.4% among participants who completed the full protocol.

By contrast, the use of objective psychophysiological measures constitutes a complementary approach to improving the accuracy of assessment in individuals with intellectual disability. In this regard, heart rate variability (HRV) has emerged as a relevant physiological indicator, as it reflects the interaction between the sympathetic and parasympathetic nervous systems in cardiovascular regulation [17]. HRV has been considered an index of the organism’s capacity to adapt to stressors and regulate psychophysiological responses, showing associations with emotional states such as anxiety, depression, and perceptions of safety [18,19]. In parallel, wearable technologies enable the monitoring of physiological parameters in naturalistic settings, thereby expanding the possibilities for the detection and monitoring of conditions related to stress, anxiety, and depression [20].

In response to these needs, the development of innovative assessment systems that enable continuous, ecological, and multimodal monitoring of mental health appears particularly relevant. MENTALSED was developed with the aim of designing a digital system to facilitate the early detection of mental health problems and to improve assessment, diagnostic, and prevention processes in individuals with intellectual disability. To this end, the system integrates information from multiple sources through an EMA design, including accessible self-reports, proxy reports from professionals, and objective psychophysiological measures, with the goal of advancing toward a more precise, contextualized, and needs-oriented assessment of the mental health of adults with intellectual disability.

## Methods

### Design and Procedure

The study used a mixed methods design, combining quantitative and qualitative procedures across the development and pilot-testing phases of the MENTALSED tool. The project began with a review of the existing literature on mental health assessment in people with intellectual disability [21] to identify available assessment tools, methodological limitations, and unmet needs in this field. Subsequently, a survey was conducted with professionals working directly with people with intellectual disability in health and social care settings with the aim of gathering their perspectives on mental health assessment and identifying specific needs in routine practice (n=31). This was carried out from January 1 to February 10, 2025.

The needs identified included the limited availability of digital tools, objective measures, and self-report instruments adapted for people with intellectual disability. On the basis of these findings, the MENTALSED assessment system was designed as a digital, multimodal tool using an EMA methodology, with self-report and proxy-report questionnaires and objective psychophysiological recordings. Two different questionnaires were developed to assess emotional, cognitive, and behavioral indicators related to mental health, one for professionals and the other for people with intellectual disability. The version for professionals also recorded medication changes and disruptive behaviors, whereas the self-report version for people with intellectual disability included sleep quality. Both questionnaires allowed complementary audio information and were designed to be completed in less than 4 minutes.

First, the questionnaire for professionals was developed using both ad hoc items and adaptations of existing standardized instruments. Once the initial item pool had been created, the questionnaire was distributed via Microsoft Forms [22] to university experts (n=6) and direct care professionals (n=10). Participants answered anonymously and evaluated the clarity, coherence, and relevance of the items and were also invited to provide qualitative suggestions regarding possible omissions or modifications. On the basis of their feedback, the questionnaire was revised and subsequently implemented on the Avicenna Research platform [23], a platform that allows questionnaires to be presented on mobile phones or tablets, sends automatic notifications, and records responses digitally, facilitating its use in natural settings and in the repeated assessments characteristic of EMA [24,25].

In parallel, the self-report questionnaire was developed by adapting ad hoc items and selected content from existing standardized instruments. Whenever possible, items were designed to be conceptually symmetrical with those included in the professional version, while ensuring accessibility for people with intellectual disability. To this end, the questionnaire was adapted into an easy-to-read format with the support of a focus group composed of people with ID (n=4), who were informed about the project and gave their consent to participate. Subsequently, a simulated version of the questionnaire was distributed via Microsoft Forms [22] to people with intellectual disability (n=16) and professionals (n=24) who answered anonymously. People with intellectual disability completed the evaluation in an easy-to-read format and were asked whether the items were important and adequately represented the construct being assessed. Professionals evaluated the clarity, coherence, and relevance of the items. In both cases, participants were invited to indicate whether any relevant aspect was missing or whether they would modify any part of the assessment. Data on the designed questionnaires were collected from professionals and people with intellectual disability from March 1 to May 30, 2025. After reviewing the responses, the necessary changes were incorporated and the self-report questionnaire was implemented using the Avicenna Research platform [23].

In addition to the questionnaires, objective psychophysiological measures were included in the evaluation to assess physiological health: HRV recorded via a chest strap and data on sleep quality and heart rate recorded via a smartwatch. Finally, all the data collected—including self-reports, third-party reports, and psychophysiological measures—were entered into a custom-developed app where they were displayed graphically in 4 dimensions: emotional well-being, general psychofunctional well-being, quality of rest, and physical well-being. Examples of this app can be seen in Figures 1 and 2.

**Figure 1.**
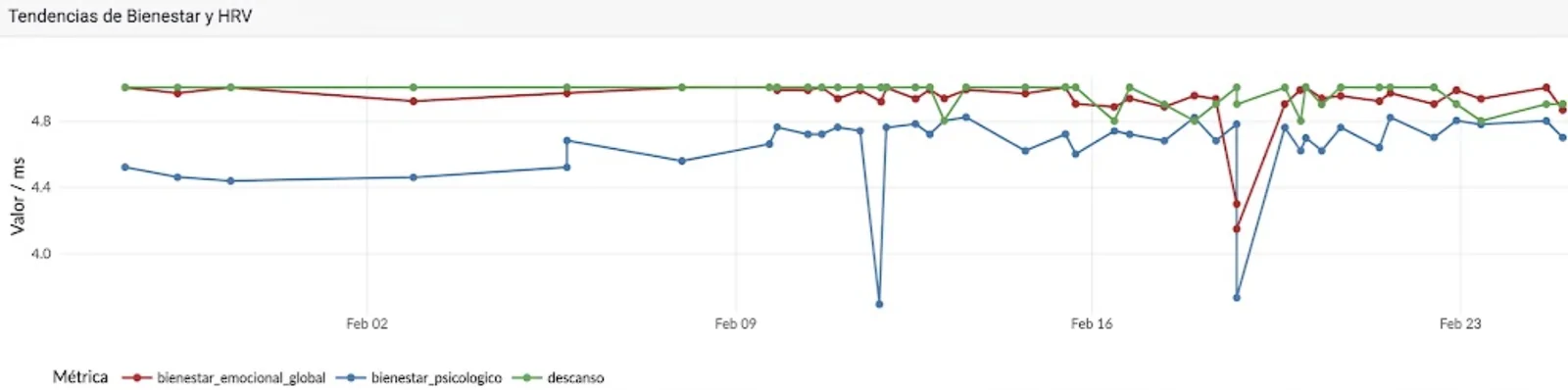
Screenshot of the app displaying longitudinal study information for professionals and people with intellectual disabilities.

**Figure 2.**
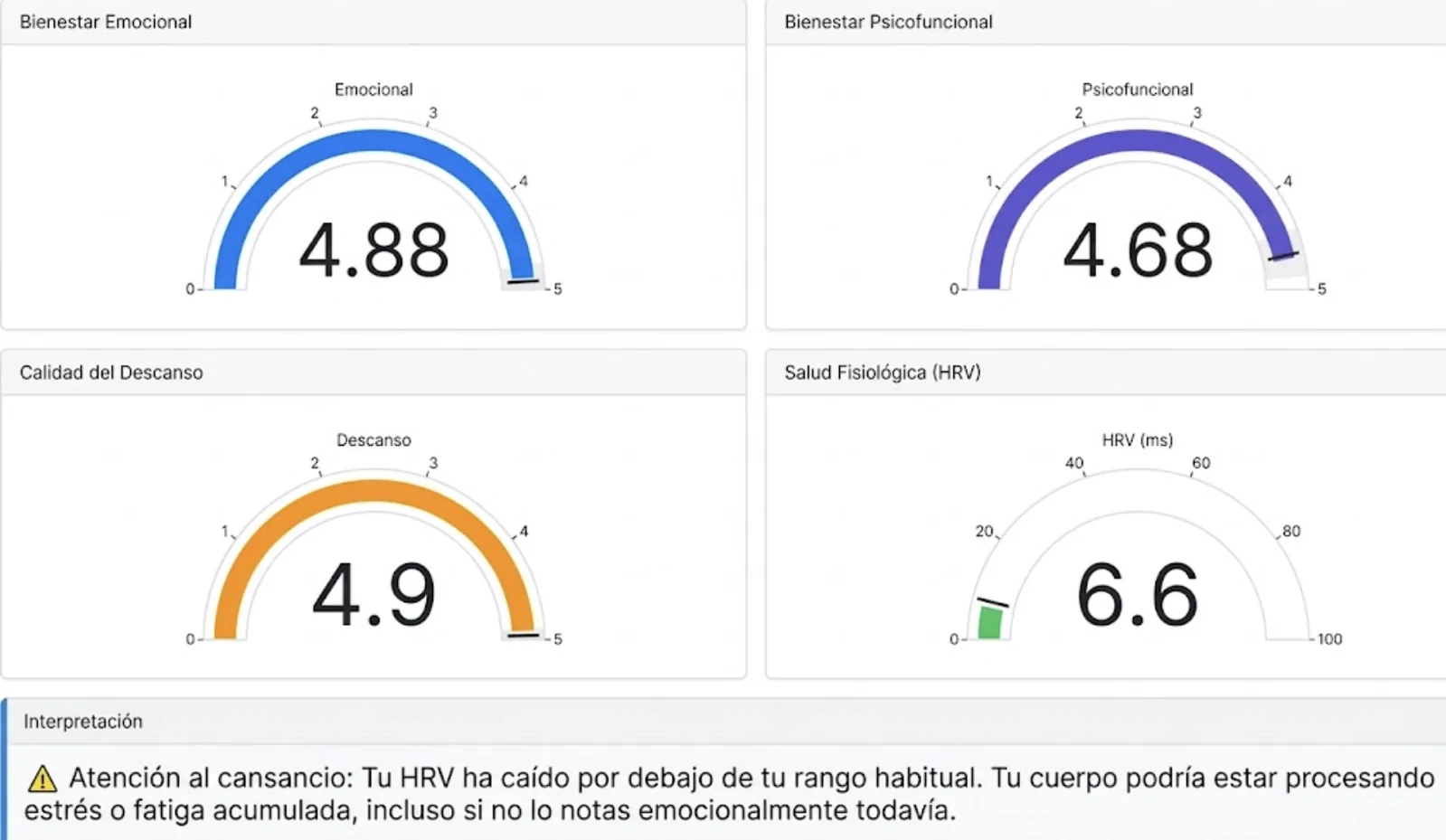
Screenshot of the app displaying daily study information for professionals and people with intellectual disabilities.

### Instruments and Variables

#### Questionnaires

Two complementary questionnaires were designed: one self-administered questionnaire for people with intellectual disability and another for professionals. Items were developed based on ad hoc questions and content adapted from standardized instruments used to assess anxiety, depression, affect, psychopathological symptoms, and behavior in individuals with intellectual disabilities. These instruments served as references for selecting and adapting the content, with priority given to those with international psychometric support and, when available, versions validated in the Spanish population or specifically designed for individuals with intellectual disabilities. These included the Patient Health Questionnaire-9 [26], the Patient Health Questionnaire-4 [27], the Generalized Anxiety Disorder-7 [28], the Glasgow Anxiety Scale for People with an Intellectual Disability [29,30], and the Glasgow Depression Scale for people with a Learning Disability and its caregiver supplement [31,32].

The proxy-report questionnaire, designed for frontline care professionals, included items on emotional well-being (9 Likert-scale items ranging from 1 [not at all] to 5 [very much]; eg, Have you experienced mood swings?); general psychofunctional well-being with information on depression (11 Likert-type items) and anxiety (4 Likert-type items); and finally, consumption of caffeine, tobacco, and caffeinated products (3 items with quantitative indicators), as well as specific records of disruptive behaviors when they occurred (4 items) and changes in medication (4 items).

The self-report questionnaire, designed for people with intellectual disability and adapted for easy reading, included a dimension on rest with 2 Likert-type items regarding general rest and the presence of nightmares; another dimension with 6 Likert-type items on emotional well-being; another dimension on general psychofunctional well-being with 6 Likert-scale items regarding the presence of anxiety and depression and 2 items to assess the presence of psychotic experiences; finally, 3 items regarding consumption of coffee or other stimulants, tobacco, and sugary products, as well as the option to add supplementary information via audio notes.

#### Objective Psychophysiological Measures

In addition to the digital questionnaires, psychophysiological and objective measurements were collected. HRV was assessed using the Polar H10 heart rate sensor [33], a chest-strap device based on electrocardiographic technology under resting conditions for 10 minutes. Additionally, heart rate, sleep indicators, and daily activity were recorded using Polar Unite smartwatches [33]. This psychophysiological component was considered optional and complementary.

### Participants

Across the different phases of the study, 71 professional contributions and 20 contributions from people with intellectual disability were obtained. In the initial phase, direct care professionals working with people with intellectual disability completed a preliminary survey (n=31). Subsequently, during the validation of the proxy-report questionnaire, university-based experts (n=6) and direct care professionals (n=10) evaluated the items. For the development of the self-report questionnaire in an easy-to-read format, an experts-by-experience panel composed of people with intellectual disability (n=4) was involved. In the subsequent evaluation phase, people with intellectual disability (n=16) and professionals (n=24) assessed the items.

### Pilot Testing

Following this period of development of the assessment tool, a pilot study will be conducted to evaluate its feasibility and functionality. The pilot testing of MENTALSED is designed to be carried out over a 2-week period, during which repeated data will be collected in naturalistic contexts using digital devices. The inclusion criteria for the pilot study are as follows: having an intellectual disability or being a direct care professional working with individuals with intellectual disabilities, having sufficient ability to use a digital device with or without support, and having sufficient cognitive ability to reflect on one’s own emotions and psychological states. The exclusion criteria are as follows: psychological or physical conditions that prevent continuous participation in data collection and an inability to understand spoken or written language. It is estimated that a minimum of 5 people with intellectual disability and 5 professionals will participate, recruited using a convenience sampling strategy based on feasibility, willingness to participate, and the existence of a regular support relationship between the professionals and the individual with intellectual disability.

The procedure will begin with an initial session in which participants will receive information about the study and provide informed consent, and professionals will complete a demographic and background form for the person with intellectual disability. The background form includes information on additional diagnoses and medication. During this first session, verbal information about the study will be provided along with an informational sheet containing the key details and contact information for the principal investigators. For people with ID, this documentation is planned to be adapted into easy-to-read format; the informational sheet will be read with them when deemed necessary, and they will be encouraged to ask questions. The consent form for participating in the study will also be adapted into an easy-to-read format and read with the person with intellectual disability if necessary. In cases where these individuals have a legal guardian or custodian, the consent form will be provided to that person for signature. Although professionals can report on more than one person with ID, for the purposes of the pilot study, each professional will be paired with a single participant with intellectual disability and will provide responses exclusively about that person throughout the study period. The pairing of a professional with an individual with intellectual disability will be done on a convenience basis, respecting what the workplace and professionals deem most appropriate (eg, if a professional regularly works with a person with intellectual disability, it may be more appropriate for that professional to respond on behalf of that person rather than on behalf of someone else they do not regularly work with). Although professionals can provide support to more than 1 person with intellectual disability in their usual work setting, each professional will provide proxy-report information exclusively about the paired participant throughout the study period. This one-to-one pairing is adopted to ensure consistency in proxy reporting and to facilitate comparison between self-report and professional proxy-report information.

To collect data, the questionnaires designed will be administered using Avicenna Research [23]. The Avicenna Research app will be installed during this first session on participants’ smartphones, when available, or on tablets provided by the research team. Participants will receive brief training on how to use the app. Objective data collection procedures will also be explained, including the use of the Polar Unite smartwatch and Polar H10 chest strap for HRV recording [33]. Devices will be provided to participants for continuous use during the study period.

Following the initial session, data collection will be carried out over 2 weeks, from Monday to Friday, resulting in 10 assessment days. The initial protocol specified that assessments through the Avicenna Research app would be completed 3 times per day by both professionals and people with intellectual disability, with automated reminders delivered to their devices. However, following consultation on the questionnaire design, this was modified to allow for only 1 assessment per day at a semiflexible time for both professionals and people with intellectual disability. Professionals will receive email notifications prompting them to complete the Avicenna Research questionnaire at the end of their workday (this can be in the morning or afternoon). People with ID will preferably complete the questionnaire in the morning, a time selected to facilitate the reporting of sleep-related variables. At the same time, a trained research technician will conduct HRV assessments under resting conditions for 10 minutes, following standardized procedures and attempting to maintain stable environmental conditions across assessment days. During the first days of data collection, the technician will also remind participants to complete the Avicenna Research questionnaires; this support is intended to be gradually withdrawn once the routine has been established. Throughout the study period, the technician will also be responsible for charging the Polar Unite [33] devices and downloading the recorded data on a weekly basis. After participating in the project, professionals and people with intellectual disability will have access to the custom-developed app, where they can view results in graphical form (Figure 3).

**Figure 3.**
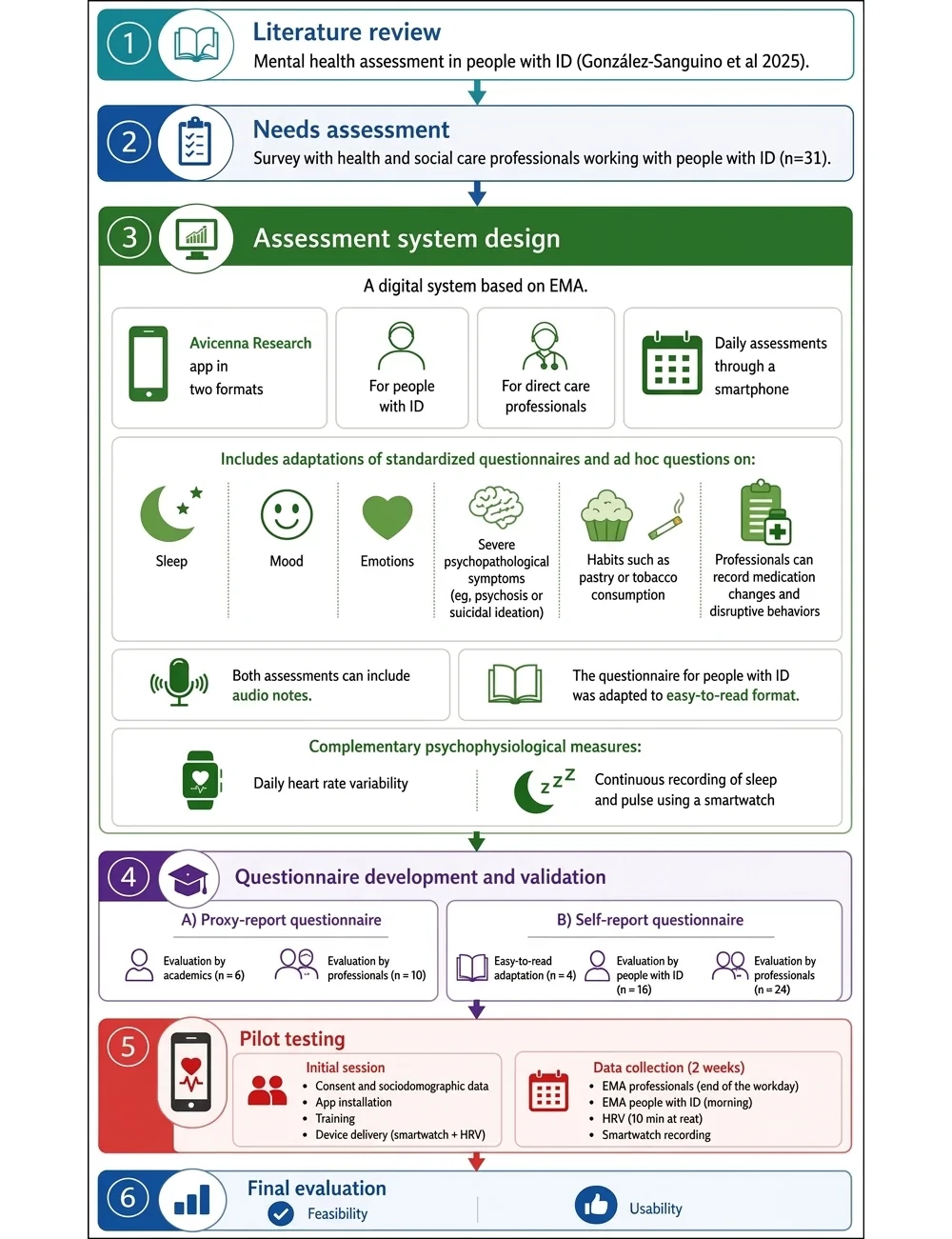
Phases of the MENTALSED study procedure. The literature review has been published [21]. ID: intellectual disability; EMA: ecological momentary assessment; HRV: heart rate variability. The image was created with the assistance of AI [34] and subsequently reviewed by the research team.

### Feasibility Outcomes and Benchmarks

The primary aim of the pilot phase is to evaluate the feasibility of implementing the MENTALSED protocol in naturalistic settings. The primary feasibility outcomes are completion, adherence, usability, acceptability, missing data, and device-wear time. Completion and adherence are defined as the proportion of scheduled EMA questionnaires completed by people with intellectual disability and professionals over the 10 assessment days. The protocol will be considered feasible if at least 70% of scheduled assessments are completed. Device-wear time is evaluated through valid physiological recordings. For the Polar Unite smartwatch, valid wear time is defined as sufficient daily recording to obtain usable heart rate, sleep-related data, and daily activity; for the Polar H10 chest strap, valid HRV assessment is defined as successful completion of the scheduled 10-minute resting recording. The benchmark for physiological feasibility is valid data on at least 70% of study days. Missing data are defined as unanswered questionnaire items, incomplete assessments, or unusable or corrupted physiological recordings, with a benchmark of less than 20% missing or unusable data. Usability and acceptability are assessed through poststudy qualitative feedback from professionals and participants with intellectual disability. This benchmark is considered met if feedback indicates that the protocol can be integrated into daily routines and no critical unresolved usability barriers are identified.

### Data Analysis

Prior to formal statistical testing, a meticulous data processing and cleaning phase will be conducted to ensure the integrity of the database. Feasibility outcomes will be summarized using descriptive statistics, including frequencies, percentages, means, SDs, medians, and ranges, as appropriate. Completion and adherence will be calculated as the percentage of completed assessments relative to the total number of scheduled assessments. Missing questionnaire data and invalid physiological recordings will be reported separately.

Following data preparation, descriptive analyses will be carried out to characterize the sample and summarize the key variables under study. These analyses will include calculating means, SDs, frequencies, and percentages to describe participants’ demographic profiles, baseline clinical characteristics, and overall adherence and completion rates for the EMA protocol.

Repeated-measures data will be analyzed using exploratory linear mixed-effects models when the number of observations, distribution of the variables, and model convergence permit. The main outcome variables will be daily emotional well-being and daily psychofunctional and psychological well-being scores derived from the EMA questionnaires. For models examining change over time, assessment day will be included as a fixed effect. For models comparing self-report and professional proxy-report information, report source will be included as a fixed effect. When physiological data are available, HRV indices, particularly root mean square of successive differences, will be examined as exploratory predictors of daily well-being outcomes. Random intercepts for the participant or dyad will be included to account for the repeated-measures structure. Given the small feasibility sample, random slopes will not be estimated unless supported by the data.

Missing questionnaire data will not be imputed for the primary feasibility analyses. Available-case analyses will be used for descriptive summaries, and mixed-effects models will be estimated using maximum likelihood procedures under the missing-at-random assumption when applicable. Missing or invalid physiological data will be treated as feasibility outcomes and reported separately.

Agreement between self-reports from people with intellectual disability and proxy reports from professionals will be examined using paired daily comparisons, graphical inspection of individual trajectories, correlation analyses, and mixed-effects models including report source as a fixed effect. These analyses will be interpreted as exploratory and will be used to assess whether both sources provide convergent or complementary information.

### Ethical Considerations

All procedures contributing to this work comply with the ethical standards of the relevant national and institutional committees on human experimentation and with the Declaration of Helsinki of 1975, as revised in 2013. All procedures involving human participants and patients were approved by the Deontological Commission of the Universidad de Valladolid (2025-CEUVa-017-H-1-Y-4). No financial compensation was provided for participating in the study. Written informed consent was obtained from all participants and, when required, from legal guardians.

## Results

The project was funded by the Ministry of Science, Innovation and Universities and the State Research Agency (MICIU/AEI) and the ERDF/EU (grant PID2023-150190OA-I00/MENTAL-SED). The needs assessment and validation phases were conducted between January and May 2025. As of manuscript submission, a total of 91 participants (71 professionals and experts and 20 individuals with intellectual disability) have been recruited and have participated in the development and validation stages. The 2-week pilot study is currently concluding data collection. Data analysis for the pilot phase is expected to begin next month, and the final results are expected to be published in 2027.

## Discussion

### Anticipated Findings

The present study describes the development and pilot testing of MENTALSED, a digital multimodal system designed to improve mental health assessment in people with intellectual disability. The system integrates EMA, self-report and proxy-report questionnaires, and psychophysiological measures with the aim of providing a more continuous, contextualized, and person-centered assessment of emotional, cognitive, and behavioral indicators.

One of the main contributions of MENTALSED is its potential to capture dynamic mental health patterns that may not be detected through traditional single time point assessments. The use of EMA allows repeated data collection in naturalistic contexts and may facilitate the early identification of emotional and behavioral changes. This is consistent with previous research highlighting the value of EMA [11] in reducing recall bias and improving ecological validity [25]. However, EMA studies must be designed carefully. Our initial design called for 3 daily assessments, but these were discarded after consulting with the professionals, as they found them unmanageable in their work. This is in line with previous studies where lower-frequency, longer-duration protocols were associated with higher compliance [16] and is consistent with evidence suggesting that completion rates in EMA-based studies may be influenced by cognitive demands and assessment burden [13,16]. In contrast, more intensive protocols resulted in lower compliance in previous studies [15].

A second relevant contribution is the combination of self-report and proxy-report information. Traditional assessment in people with intellectual disability often relies heavily on external informants, which may limit the detection of internal experiences [35]. The lack of cognitively accessible self-report measures contributes to underrecognition [35]. Accordingly, the development of an accessible self-report format in MENTALSED is consistent with evidence-based recommendations [36]. At the same time, the proxy-report questionnaire provides complementary information. Comparing both sources may help identify convergences and discrepancies, reinforcing the need for multimethod approaches [14]. Recent evidence also suggests that proxy measurement of internal states presents limitations, reinforcing the need to combine proxy reports with self-report data [37]. Moreover, this multimodal structure may help reduce the risk of diagnostic overshadowing [7].

The inclusion of psychophysiological measures represents an additional strength. HRV has been associated with psychophysiological regulation and emotional states in daily life [19]. In people with intellectual disability, HRV monitoring has been explored as a potential marker of changes in behavioral excitation [38]. This multimodal approach is aligned with research combining physiological signals and subjective data [18,20]. However, physiological indicators from wearable devices should be interpreted cautiously alongside self-report and behavioral information [39]. The use of digital tools may also facilitate data storage, management, and visualization, generating individualized profiles. This could support decision-making processes in care contexts. This is consistent with work highlighting the value of mobile technologies [12] and continuous monitoring [14]. However, digital familiarity does not imply immediate usability.

### Conclusions

MENTALSED represents a promising digital and multimodal approach. By integrating accessible self-report, professional proxy-report, EMA, and psychophysiological data, the system may contribute to more accessible, continuous, and person-centered assessment models.

## Supplementary material

10.2196/106072 Peer Review Report 1 Peer review report by Spanish Ministry of Science, Innovation and Universities/State Research Agency (MICIU/AEI) and the European Regional Development Fund (ERDF/EU), grant PID2023-150190OA-I00/MENTAL-SED.

10.2196/106072 Peer Review Report 2 Translation of the peer review report.

## Abbreviations

EMA: ecological momentary assessment
HRV: heart rate variability
