# Supplementary material for: A Digital System to Assess Mental Health in People With Intellectual Disability (MENTALSED): Protocol for a Development and Pilot Study
Source: JMIR Res Protoc. 2026 Sep 30;15:e106072. doi: 10.2196/106072 (PMC13626399; doi:10.2196/106072)
Supplement: Supplementary file 1 [file resprot-v15-e106072-s001.pdf]

	**Proyectos de Generación de Conocimiento 2023<br>Investigación Orientada Tipo A**
**Referencia:**	PID2023-150190OA-I00
**Área:**	Psicología
**Subárea:**	Psicología
**Investigador/a principal**	RODRIGUEZ MEDINA, JAIRO GONZALEZ SANGUINO / CLARA
**Título:**	SALUD MENTAL EN PERSONAS CON DISCAPACIDAD INTELECTUAL. EVALUACION, DIAGNOSTICO Y PREVENCION MEDIANTE UN SISTEMA DE EVALUACION DIGITAL.

De acuerdo con los principios de discrecionalidad técnica e independencia, este informe de valoración científico técnica se elabora teniendo en cuenta el consenso adoptado en la comisión técnica, una vez analizadas las valoraciones cualitativas de los informes de experto y las opiniones de los expertos de la comisión, y a la vista del conjunto de proyectos presentados a cada área temática. Por ello, este informe no tiene por qué asumir, en todos sus términos, las valoraciones aportadas por los expertos en sus informes, que pueden ser discrepantes y que se reconocen inevitables y legítimas siempre que no incurran en errores técnicos o fácticos graves y manifiestos.

PROYECTOS DE GENERACIÓN DE CONOCIMIENTO 2023

MODALIDAD: INVESTIGACIÓN ORIENTADA TIPO A

INFORME DE VALORACIÓN CIENTÍFICO TÉCNICA - COMISIÓN TECNICA

PARTE 1

CRITERIOS DE EVALUACIÓN

1.Calidad y viabilidad de la propuesta

UMBRAL 30

Puntuación de 0 a 40: 34.7

1.1. Calidad de la propuesta

La Comisión considera que la propuesta se adecúa a la finalidad de la convocatoria, abordando un tema tan relevante como es la mejora de la salud mental de las personas con discapacidad intelectual. Para ello, se pretende desarrollar una herramienta de evaluación digital. Los objetivos están bien definidos y se encuentran bien fundamentados. Como sugerencias de mejora de la propuesta, se recomienda detallar mejor el procedimiento para mantener y actualizar la herramienta digital una vez finalizado el proyecto.

Puntuación 0 a 25: 22.0

1.2 Viabilidad de la propuesta

Los IPs cuentan con una trayectoria relevante en el ámbito de la discapacidad intelectual, lo que avala la viabilidad de la propuesta. La planificación temporal del proyecto es adecuada y las actividades para la consecución de los objetivos están bien definidas. Como recomendación, convendría detallarse el plan de formación para profesionales. También, se recomienda aportar más información sobre el tamaño muestral y las características de la muestra.

Puntuación 0 a 15: 12.7

2. Calidad y trayectoria de los componentes del proyecto

UMBRAL 20

La trayectoria tanto de los IP como del equipo se considera relevante, estando demostrada la capacidad de liderazgo de los investigadores principales. La Comisión valora positivamente las colaboraciones y publicaciones conjuntas previas entre los miembros del equipo, así como las acciones de internacionalización. La Comisión no ha priorizado el proyecto para optar a un contrato predoctoraI para la formación de doctores, dada la valoración alcanzada y el número de contratos disponibles.

Puntuación 0 a 30: 24.4

3. Impacto científico, económico y social esperado de los resultados

UMBRAL 10

El impacto científico del proyecto es alto, dado que la creación de una herramienta de evaluación digital puede suponer un avance en el conocimiento en el campo de la discapacidad intelectual. El impacto social también es considerable, al mejorar la vida de las personas. La Comisión considera que el plan de transferencia está bien diseñado y que el plan de difusión de los resultados es adecuado.

Puntuación 0 a 20: 17.6

4. Adecuación del presupuesto asociado

El presupuesto está en general bien justificado, aunque se considera excesivo el gasto en personal destinado a la recogida de datos.

Puntuación 0 a 10: 6.8

Valoración Global

Puntuación de 0 a 100: 83.5

PARTE 2

OTROS ASPECTOS A CONSIDERAR

A) Aspectos relacionados con zonas polares o campañas oceanográficas(Cumplimentar solo en los proyectos que proceda)

No procede.

B) Condiciones específicas para la ejecución de determinados proyectos (Cumplimentar solo en los proyectos con aspectos relacionados con las condiciones o implicaciones recogidas en el Anexo IV de la convocatoria).

No procede.

INFORME-EXPERTO 1

PARTE 1

CRITERIOS DE EVALUACIÓN

1.Calidad y viabilidad de la propuesta

1.1 Calidad de la propuesta

La presente propuesta se adecúa plenamente a la modalidad de la convocatoria, investigación orientada, siendo un proyecto que pretende abordar el diseño, desarrollo e implementación de una herramienta de evaluación integral y digitalizada para evaluar la salud mental en personas con discapacidad intelectual. Asimismo, se ajusta a la prioridad temática "Salud", trabajando lateralmente la prioridad temática "Sociedad inclusiva" (por las claras repercusiones que para la inclusión tiene el abordaje de la salud mental en esta población) a través de herramientas, tecnologías y soluciones digitales para la salud y cuidados (prioridad temática incluida en el Plan Estatal de Investigación Científica, Técnica y de Innovación 2021-2023). La propuesta es relevante y original, ya que en nuestro país no contamos con una herramienta para registrar de modo fácil y preciso aquellos indicadores de problemas de salud mental en personas con discapacidad intelectual (PDI), contribuyendo así a la precisión del diagnóstico y, probablemente, también a la detección e intervención precoz. No expone una hipótesis de partida, por lo que no podemos valorar si esta es válida y original. Sería recomendable (y necesario en esta convocatoria) exponer la dirección de los resultados que se espera encontrar, al amparo de la evidencia previa existente. Desarrolla un marco teórico y unas preguntas de investigación que guían los objetivos, siendo estos relevantes, y estando claramente definidos y fundamentados. Es un proyecto interdisciplinar, donde se incluyen profesionales y expertos de diferentes ámbitos, como es psicología, pedagogía, trabajo social e informática y telecomunicaciones.

1.2 Viabilidad de la propuesta

Los resultados y contribuciones previos del equipo participante y de los IPs avalan la viabilidad de la propuesta, destacando la gran experiencia en el trabajo con personas con DI, en el desarrollo y validación de instrumentos de evaluación con esta población, así como en los análisis propuestos. También han realizado diversas acciones de transferencia a la sociedad, como es el diseño de un sistema de evaluación integral para la Comunidad de Madrid. En cuanto a la metodología, los objetivos se presentan con claridad y precisión; y las actividades plantadas se consideran adecuadas a los mismos. El objetivo 1 es realizar una revisión sistemática de la literatura científica sobre instrumentos empleados para prevenir la aparición de problemas de salud mental en personas con DI, y su metodología es adecuada. Con respecto al objetivo 2 (fase 1), no se realiza una descripción del marco muestral ni un análisis del tamaño de la muestra necesario, por lo que no podemos conocer si el proyecto es viable y ni si será representativo. Para el objetivo 3 (fase 2) tampoco se hace un análisis del marco muestran ni un análisis del tamaño de la muestra. En este objetivo, se menciona que se administrará un instrumento de observación sistemática y pruebas psicométricas estandarizadas, pero no especifica qué pruebas concretas se administrarán, por lo que no podemos conocer la pertinencia de las mismas. Quizás se refiere a la administración de los instrumentos detectados y reconocidos como "no válidos" en la fase 1, pero entendemos que esto no puede ser así (el diseño de un instrumento de evaluación tiene que partir de la validez de otros instrumentos, no de mejorar los considerados no válidos). Sobre los objetivos 4, 5, 6 y 7, tampoco se realiza una descripción del marco muestral ni un análisis del tamaño de la muestra necesario; por lo que no podemos conocer si la propuesta es viable en relación con el marco muestral, ni si será suficiente. Establecen las etapas críticas junto al posible plan de contingencias. Sin embargo, no valoran que evaluar a 600 personas con DI pueda suponer un punto crítico, y sin embargo valoramos que pueden tener problemas con el acceso a la muestra. Otro punto crítico es el proceso de digitalización, que según lo mencionado estará a cargo de una empresa externa que tendrá 6 meses para ejecutarlo. Quizás esta temporalización resulte algo ajustada.

2. Calidad y trayectoria de los componentes del proyecto

Los resultados y contribuciones previos del equipo participante y de los IPs avalan la viabilidad de la propuesta. Así, podemos destacar una importante trayectoria científica con publicaciones con impacto publicadas en el campo de la DI. Algunos miembros del equipo han sido responsables de la implementación de la primera encuesta nacional sobre el estigma hacia las personas con DI. Otro aspecto a destacar es la experiencia en el desarrollo y validación de instrumentos de evaluación para personas con DI. Además, una de las investigadoras es responsable del diseño de un sistema integral de evaluación de las personas con DI en centros dependientes de la Comunidad de Madrid. Sin embargo, son escasas las colaboraciones mutuas; y algunas de publicaciones son MDPI o Frontiers. Sobre las posibilidades de internacionalización, si bien plantean la posibilidad de preparar diversas propuestas de investigación derivadas del proyecto para convocatoria ERASMUS+, no cuentan con ningún miembro internacional que facilite esta iniciativa.

3. Impacto científico, económico y social esperado de los resultados

La propuesta cuenta con un impacto científico alto, al explorar la salud mental de las personas con DI, campo de estudio altamente novedoso. A nivel social supone el desarrollo de una herramienta que podrá tener un impacto considerable en el ámbito de la discapacidad, ya que estará informatizada y se ofrecerá de modo gratuito a entidades y profesionales que trabajan con esta población. Además, el proyecto contribuirá a mejorar las herramientas de diagnóstico de los trastornos de salud mental, lo que podrá repercutir en una detección -y consecuente intervención- precoz, y esto as su vez en una reducción del gasto sanitario y de medicamentos. Cuenta con un plan de divulgación de resultados, centrado en un informe técnico, materiales divulgativos accesibles, y publicaciones en medios de divulgación (no señala cuáles). El plan de transferencia se centra en la difusión de los resultados en congresos, workshop, y publicaciones científicas. Creemos, al leer la propuesta, que dicho plan se solapa con la propuesta de difusión científica. En esta mencionan 15 artículos, pero no describen los hitos o temática concreta sobre la que versarán cada uno de estos, ni la propuesta de revistas. Estimamos que el presente proyecto puede contar con publicaciones de interés e impacto, pero probablemente 15 artículos sean demasiados para los objetivos del mismo. Valoramos el interés mostrado por los IPs en que los resultados del proyecto lleguen a pacientes, familiares y profesionales; destacando la inclusión de actividades de apoyo científico-técnico a entidades del tercer sector que requieran conocimientos expertos para el uso del sistema digital de evaluación o bien para la planificación de estudios aplicados, y el interés por desarrollar material de formación dirigido a investigadores, profesionales, familias y personas con DI interesados. Cuenta con un adecuado plan de gestión de los resultados del proyecto, destacando que los resultados serán localizables, accesibles, interoperables y se podrán reutilizar en el contexto de la investigación científica (principios FAIR). La propuesta contempla la inclusión de la perspectiva de género en los objetivos, actividades y metodología. Recomendamos apoyar dicha inclusión con el marco teórico, ya que es amplia la literatura que evidencia las desigualdades de género en el ámbito de la salud mental y de la discapacidad. El ámbito de la discapacidad es intrínseco a la propia propuesta.

4. Adecuación del presupuesto solicitado

Las partidas están suficientemente detalladas y justificadas. Solicita una partida de 6000Euros para la compra de bandas Polar H10 para la recogida de datos de la variabilidad de la frecuencia cardiaca (3000 euros aprox.), para material informático (2000) y para otros gastos asociados (1000 euros), pero no detalla qué gastos se incluyen en esta partida. También se incluye una partida para publicaciones en abierto. En este sentido, en la presente convocatoria se recomienda utilizar repositorios gratuitos, como los que tienen la mayoría de universidades o el Digital.CSIC; y no partidas específicas con estos gastos.

PARTE 2

OTROS ASPECTOS A CONSIDERAR

A) Capacidad Formativa

Se detalla la formación que se proporcionará desde el programa de doctorado en Investigación Transdisciplinar en Educación en la Universidad de Valladolid, que incluye actividades formativas sobre metodología de investigación, asistencia a congresos y publicación de -al menos- un artículo científico. Enumeran 16 tesis doctorales defendidas en los últimos años, dos de ellas sobre el tema de discapacidad; el resto especialmente centradas en el funcionamiento ejecutivo y neuroeducación. No describen el desarrollo científico y/o profesional de los doctores egresados del equipo de investigación.

B) Aspectos relacionados con zonas polares o campañas oceanográficas

NA

C) Condiciones específicas para la ejecución de determinados proyectos

Describe las consideraciones específicas que tendrán en cuenta al trabajar con seres humanos.

INFORME-EXPERTO 2

PARTE 1

CRITERIOS DE EVALUACIÓN

1.Calidad y viabilidad de la propuesta

1.1 Calidad de la propuesta

La propuesta se adecúa a las características y finalidad de la convocatoria, enfocándose en la mejora de la salud mental de las personas con discapacidad intelectual (PCDI) mediante el desarrollo de una herramienta digital de evaluación y diagnóstico. Esta propuesta está alineada con la prioridad temática de Salud y Sociedad Inclusiva, como se detalla en el Plan Estatal de Investigación Científica, Técnica y de Innovación 2021-2023. Específicamente, se enfoca en herramientas y soluciones digitales para la salud y cuidados, así como en promover una sociedad inclusiva al abordar las barreras que enfrentan las PCDI. La propuesta es altamente relevante debido a la significativa necesidad no cubierta de herramientas diagnósticas adaptadas para PCDI. La hipótesis de partida es válida y original, apuntando a que las PCDI pueden tener psicopatologías comórbidas con mayor frecuencia que la población general, pero su diagnóstico es complicado debido a la falta de herramientas adaptadas. La propuesta de desarrollar una herramienta digital que combine la evaluación de problemas de comportamiento, salud mental y bienestar emocional es innovadora y aborda directamente esta necesidad. Los objetivos están claramente definidos. Los objetivos generales, que incluyen la profundización en el conocimiento de los problemas de salud mental en PCDI y el desarrollo de una herramienta digital de evaluación, son ambiciosos y relevantes. También son realistas dado el plan de trabajo y la metodología interdisciplinar propuesta. Se basan en metodologías comprobadas y en la experiencia previa del equipo investigador. La propuesta justifica adecuadamente su enfoque multidisciplinar. Involucra a profesionales de diferentes disciplinas, lo que permite abordar la problemática desde diferentes perspectivas. La colaboración con diversas universidades y entidades tanto a nivel nacional como internacional fortalece la viabilidad y potencial de impacto del proyecto. No obstante, la propuesta tiene algunas debilidades. El éxito del proyecto depende en gran medida del desarrollo y adopción de la herramienta digital propuesta. Cualquier retraso o fallo en la implementación tecnológica podría comprometer el logro de los objetivos. La metodología interdisciplinar puede presentar problemas en la coordinación e integración de diferentes disciplinas y enfoques. Esto podría resultar en retrasos o dificultades en la ejecución del proyecto. Aunque el proyecto aborda una necesidad específica, la variabilidad en las PCDI puede hacer que los resultados y las herramientas desarrolladas no sean aplicables a todas las subpoblaciones dentro de este grupo. La personalización y adaptabilidad de la herramienta a diferentes contextos y niveles de discapacidad conlleva una dificultad considerable. La propuesta no detalla en profundidad los planes para la sostenibilidad y escalabilidad de la herramienta una vez finalizado el proyecto. Esto incluye cómo se mantendrá y actualizará la herramienta y cómo se garantizará su adopción a largo plazo por parte de las entidades y profesionales del sector. La propuesta confía en obtener información durante el proyecto para definir la herramienta, en lugar de basarse en la literatura previa y en desarrollos existentes. Sería conveniente tener una definición más clara y basada en investigaciones previas para asegurar que la herramienta se desarrolla sobre una base comprobada.

1.2 Viabilidad de la propuesta

Los miembros del equipo investigador, y en particular los dos IPs, cuentan con una trayectoria relevante en el ámbito de la psicología clínica y la salud mental, especialmente en contextos de discapacidad intelectual. Ambos IP tienen publicaciones significativas y experiencia en proyectos relacionados, lo cual avala la viabilidad de la propuesta. Los objetivos específicos están claramente definidos y se alinean con la duración del proyecto y la composición del equipo. Los objetivos están organizados temporalmente de manera lógica, permitiendo una progresión de las actividades desde la revisión sistemática de la literatura hasta el desarrollo y validación de la herramienta digital. La planificación temporal parece adecuada para la ejecución del proyecto. No obstante, la dirección de un equipo interdisciplinario conlleva dificultades que hacen recomendable un plan de coordinación más desarrollado. Las actividades descritas para alcanzar los objetivos propuestos son adecuadas y están bien estructuradas. La propuesta demuestra comprensión de las dificultades metodológicas y ofrece soluciones para superarlas. Sin embargo, la recolección de datos empíricos de calidad, especialmente de una población vulnerable como las PCDI, puede presentar problemas éticos y logísticos, y la dependencia del desarrollo tecnológico propuesto puede representar un riesgo, ya que problemas técnicos imprevistos podrían comprometer el logro de los objetivos. Sería beneficioso incluir especificaciones técnicas más detalladas desde el inicio, basadas en la literatura previa. En cualquier caso, el proyecto identifica las etapas críticas que podrían afectar a la viabilidad del plan de trabajo, como el desarrollo tecnológico de la herramienta digital y la recogida de datos empíricos. El plan de contingencia propuesto es adecuado en líneas generales y contempla alternativas razonables para mitigar algunos de los posibles riesgos, como ajustes en el cronograma, reestructuración de actividades y recursos adicionales en caso de retrasos tecnológicos o dificultades en la recogida de datos. En otros riesgos las medidas previstas no parecen suficientes, confiando únicamente en su baja probabilidad de ocurrencia.

2. Calidad y trayectoria de los componentes del proyecto

Se trata de un equipo numeroso y con buena trayectoria. El IP1 ha publicado numerosos artículos y ha participado en varios proyectos de investigación competitivos. Su experiencia en proyectos interdisciplinares y su historial de publicaciones lo avalan como un líder adecuado para este proyecto. La IP2 tiene experiencia en la investigación de la salud mental y el estigma. Ha realizado numerosas publicaciones sobre esos temas, y su trabajo ha sido reconocido con premios de investigación. Su experiencia en salud mental y estigmatización, junto con su historial de colaboración en proyectos, también la avalan para liderar este proyecto. El resto del equipo de investigación cuenta con miembros especializados en psicología, pedagogía, práctica social, informática y telecomunicaciones. Esta diversidad de conocimientos es adecuada para el desarrollo del proyecto. Existen evidencias de colaboraciones previas entre los integrantes del equipo, como coautoría en publicaciones y participación conjunta en proyectos de investigación. Esta cohesión y experiencia conjunta fortalecen la viabilidad del proyecto. Mantienen colaboraciones con grupos y entidades internacionales en Europa y América, lo que se refleja en proyectos conjuntos y coautorías de publicaciones.

3. Impacto científico, económico y social esperado de los resultados

El proyecto puede tener un impacto científico-técnico relevante. Aborda una necesidad en la evaluación y diagnóstico de la salud mental en personas con discapacidad intelectual (PCDI), un área que ha sido insuficientemente investigada y carece de herramientas diagnósticas adecuadas. El desarrollo de una herramienta digital innovadora que combina la evaluación de problemas de comportamiento, salud mental y bienestar emocional puede contribuir al avance del conocimiento en este campo y establecer nuevos estándares en la evaluación de la salud mental en PCDI. El impacto social de la propuesta es también potencialmente relevante. La herramienta digital desarrollada puede mejorar la calidad de vida de las PCDI al facilitar un diagnóstico más preciso y temprano de problemas de salud mental, permitiendo intervenciones más efectivas. Esto no solo beneficia a las PCDI y sus familias, sino que también puede reducir el estigma y promover la inclusión social. Económicamente, la herramienta puede ahorrar recursos al mejorar la eficiencia de las evaluaciones y reducir la necesidad de tratamientos más intensivos y costosos a largo plazo. El plan de transferencia y valorización de los resultados está bien diseñado. Incluye la colaboración con profesionales del tercer sector y entidades que trabajan directamente con PCDI, asegurando que la herramienta desarrollada será relevante y útil en contextos reales. La participación de usuarios finales, como profesionales de atención directa y familiares, en el diseño y validación de la herramienta, fortalece su aplicabilidad y aceptación. La propuesta presenta un plan de difusión y divulgación adecuado, dirigido tanto a la comunidad científica como a la sociedad en general. Incluye la publicación de resultados en revistas científicas, presentación en conferencias internacionales (en este punto sería recomendable una mayor concreción), y actividades de divulgación dirigidas a profesionales de la salud, educadores y el público en general. Sin embargo, las previsiones de producción científica indicadas en la propuesta parecen poco realistas. La expectativa de 20 publicaciones en revistas indexadas, 10 en otras revistas, 2 libros, 10 capítulos de libro, 20 publicaciones en congresos internacionales y 20 en congresos nacionales en un solo proyecto es excesivamente ambiciosa y no se alinea con la producción típica de proyectos de esta envergadura ni con el historial de los investigadores del equipo. Sería más prudente ajustar estas cifras a un número más realista, garantizando así la calidad de las publicaciones. La propuesta incluye un plan adecuado de gestión de datos de investigación, y aborda adecuadamente la dimensión de género, especialmente en el ámbito de la discapacidad y otras áreas de inclusión social. Se considera la inclusión de mujeres y personas de edad avanzada dentro de las PCDI, y se proponen actividades y metodologías específicas para abordar las necesidades particulares de estos subgrupos.

4. Adecuación del presupuesto solicitado

Se solicita la contratación de personal para apoyar al equipo en la recogida de datos. Dato el extenso número de miembros del equipo de investigación y del equipo de trabajo, no acaba de apreciarse la necesidad de apoyo en esas tareas. Es necesario especificar el material informático que se incluye en el apartado de inventariable. El presupuesto para la participación en congresos y publicaciones parece insuficiente, dado el elevado número de congresos en los que se quiere participar y el número de publicaciones previstas.

PARTE 2

OTROS ASPECTOS A CONSIDERAR

A) Capacidad Formativa

El programa de formación previsto es adecuado, aunque sería conveniente especificar en mayor medida las actividades concretas de formación, más allá de las programadas en el marco del programa de doctorado. Los IPs no tienen todavía una experiencia amplia en la dirección de tesis, pero sí el equipo de investigación en su conjunto, con una larga lista de doctorandos que han pasado por el grupo. Sería conveniente también prever en el programa de formación la realización de estancias en otros centros, tanto nacionales como internacionales.

B) Aspectos relacionados con zonas polares o campañas oceanográficas

.

C) Condiciones específicas para la ejecución de determinados proyectos

La propuesta justifica adecuadamente los procedimientos y protocolos que prevé aplicar

INFORME-EXPERTO 3

PARTE 1

CRITERIOS DE EVALUACIÓN

1.Calidad y viabilidad de la propuesta

1.1 Calidad de la propuesta

- El Proyecto se adecúa a la convocatoria y sus modalidades (Acción Estratégica 1-Salud). También se ajusta a la prioridad temática (Salud-Herramientas, tecnologías y soluciones digitales para la salud y cuidados). Ambos puntos están claramente explicados al comienzo de la Memoria. También se vincula a la Acción estratégica 2 (Cultura , creatividad y Sociedad inclusiva). - El Proyecto es relevante e innovador. La hipótesis está bien estructurada y es original en su planteamiento. Los objetivos son claros y realistas... Y muy ambicioso, entre otras cosas, por el número de participantes requeridos para conseguir los distintos los distintos objetivos planteados. - El Proyecto es claramente multidisciplinar y la inclusión de las diferentes áreas de conocimiento (con sus repectivos especialistas dentro del equipo de investigación) están claramente justificadas en la metodología y son relevantes para la consecución de los objetivos propuestos.

1.2 Viabilidad de la propuesta

- El Proyecto es claramente viable dada la trayectoria del equipo investigador, que demuestra la experiencia de los investigadores en el tema del Proyecto. Dicha trayectoria está avalada por publicaciones de impacto y proyectos de investigación financiados. - Objetivos específicos bien explicados y consistentes con la cronología propuesta para desarrollar el Proyecto. El equipo investigador es competente y adecuado a la consecución de los objetivos. Hay equilibrio de género. - El plan de contingencia es correcto, aunque no aborda la posibilidad de baja participación de los grupos de individuos implicados (pacientes, profesionales).

2. Calidad y trayectoria de los componentes del proyecto

- El IP1 tiene un buen CV en el que demuestra experiencia en el tema del Proyecto que corrobora con un respetable número de publicaciones y proyectos de investigación financiados, alguno de ámbito europeo. Su capacidad formativa está avalada con la dirección de 4 Tesis Doctorales. No hay reseñas de transferencia a la industria o sociedad. Aparentemente el IP1 debe tener capacidad para el liderazgo de este Proyecto. - El IP2 tiene también un buen CV, no tan extenso como el IP1 (lógico por diferencia de edad), pero que complementa el del IP en el ámbito de la Psicología. Ha participado en actividades colaborativas docentes de ámbito internacional (OPS). Capacidad formativa que incluye la co-dirección de una Tesis Doctoral, de TFMs, así como docencia en la Universidad Complutense de Madrid y en la Universidad de Valladolid. - Las aportaciones de los miembros del equipo investigador, en conjunto, son importantes y sus trayectorias avalan la consecución de los objetivos propuestos en el Proyecto. Destacan dos investigadores (MM y MJIM) con uns CVs de excelencia en áreas de conocimiento relevantes para el Proyecto. - Las colaboraciones internacionales del equipo investigador son limitadas.

3. Impacto científico, económico y social esperado de los resultados

- El impacto científico y económico puede ser relevante, pero lo serán más el impacto social si se cumplen los objetivos del proyecto, lo que parece probable valorando la propuesta y el equipo investigador. - El plan de transferencia de resultados es excelente, realista y está muy bien explicado en la memoria. Los usuarios finales participan en varios ámbitos metodológicos. - El plan de difusión/divulgación es correcto y realista e incluye el manejo de los datos de investigación en relación a los objetivos conseguidos, especialmente su aplicación a los destinatarios en la sociedad. - En el ámbito de género, la propuesta incluye menciones específicas a la igualdad tanto en los participantes (equipo investigador) como en los destinatarios de la investigación (personas con discapacidad).

4. Adecuación del presupuesto solicitado

- El presupuesto es adecuado a los objetivos planteados y está detallado en los diferentes tipos de gastos.

PARTE 2

OTROS ASPECTOS A CONSIDERAR

A) Capacidad Formativa

N/A

B) Aspectos relacionados con zonas polares o campañas oceanográficas

N/A

C) Condiciones específicas para la ejecución de determinados proyectos

N/A

INFORME-EXPERTO 4

PARTE 1

CRITERIOS DE EVALUACIÓN

1.Calidad y viabilidad de la propuesta

1.1 Calidad de la propuesta

Apreciados investigadores, Gracias por darme la oportunidad de revisar su interesante proyecto. El proyecto de investigación presentado se adecua perfectamente a las características y a la finalidad de la modalidad seleccionada (Salud y Sociedad Inclusiva). Las personas con discapacidad intelectual (DI) tienen un riesgo mayor de presentar trastornos de salud mental mayores que en población general, claramente argumentado y justificado. Por tanto, la necesidad de evaluar y de disponer de instrumentos para su evaluación lo antes posible, es una necesidad clara que debe ser abordada para permitir mejorar la salud de las personas con discapacidad intelectual, su calidad de vida y, en definitiva, la inclusión en nuestra sociedad. Estos instrumentos deben ser válidos, fiables y funcionales en su aplicación, especialmente para los usuarios que los van a usar. Ayudar a los distintos profesionales que atienden a las personas con DI a diagnosticar Trastornos Mentales, no deja de ser un reto altamente necesario. El propósito de disponer de una herramienta automatizada de registro es muy novedoso y, a la vez, útil y ajustado en los contextos en los que se van a usar (centros de día, centros especiales de empleo y residencias). El objetivo general es claro, al igual que los objetivos específicos. El proyecto está muy bien delimitado en fases y en cada objetivo, se describe la metodología que se usará a fin de alcanzar cada uno de ellos. Se proponen distintas aproximaciones metodológicas para alcanzar los distintos objetivos, complementándose unos a otros para alcanzar el objetivo general de la propuesta. Los objetivos son realistas y ajustados.

1.2 Viabilidad de la propuesta

Claramente las contribuciones previas del equipo participante avalan la viabilidad de la propuesta. Como se ha comentado, cada uno de los seis objetivos propuestos están muy claros y avalados por metodologías acordes a los fines propuestos. La temporalidad es adecuada. El plan de contingencia es adecuado. Especialmente importante es la colaboración con las entidades participantes a priori. Parece que se ha contactado con algunas entidades del tercer sector, aunque, creo claramente que se deberían contactar con alguna más, en caso de baja o no participación de alguna de ellas. Debería ampliarse en qué consistiría el plan de formación para los profesionales y cómo se garantizaría el aprendizaje de los contenidos y su puesta en práctica.

2. Calidad y trayectoria de los componentes del proyecto

El equipo de investigación está formado por distintos investigadores de la Universidad de Valladolid pertenecientes a un Grupo de Investigación Reconocido. Se reconoce el trabajo previo del equipo investigador con Plena Inclusión, entidad claramente reconocida en España y que agrupa distintos profesionales y familias de personas con discapacidad intelectual. El informe que presentan sobre un estudio realizado en 2021 es completo y avala la capacidad del equipo para trabajos en colaboración con entidades del tercer sector. El Investigador Principal del equipo tiene reconocida un buen perfil académico y de investigación con amplias publicaciones sobre la temática que se presenta. El Segundo Investigador Principal no presenta un perfil de investigación tan amplio ni extenso, aunque coincidente en la temática que se demanda en su investigación. El equipo, en general, ha publicado numerosos artículos tanto nacionales como internacionales, así como capítulos de libros y distintas comunicaciones a congresos, tanto nacionales como internacionales. No nos cabe la menor duda de su valía para liderar y llevar adelante el proyecto con suficientes garantías. El resto de los miembros del equipo comparten también intereses comunes y un buen currículum. A destacar la colaboración con otras universidades (Universidad Complutense de Madrid, Universidad de Zaragoza, Universidad Ramón Llull, Universidad Nacional de Mar del Plata) que puede enriquecer claramente el proyecto. Se menciona como merito la colaboración continuada con Plena Inclusión con iniciativas de aprendizaje y Servicio previas a este proyecto de investigación.

3. Impacto científico, económico y social esperado de los resultados

El proyecto de investigación que se presenta tiene importantes beneficios en el ámbito de la salud mental de las personas con discapacidad intelectual. Saber la prevalencia y tipos de trastornos mentales, como detectarlos a tiempo y abordarlos, en si mismo, es un avance importante y con un claro impacto social y comunitario que permitiría el desarrollo de nuevas políticas de actuación para este colectivo. Creemos que el proyecto tiene mayor impacto a nivel nacional que internacional, aunque el desarrollo de una herramienta digital como la que se plantea en el proyecto, puede servir de punto de inicio o comparativa con otros países. Por otro lado, el impacto social para la inclusión de las personas con discapacidad intelectual es claro ya que va a permitir ahondar en este colectivo y su problemática de trastornos mentales asociados a esta condición y que deben tratarse adecuadamente y eficientemente. A nivel económico no está tan claro que pueda representar una ganancia si no va acompañado de suficientes cambios a nivel de la salud pública en general. En relación al plan de comunicación científica e internacionalización de los resultados, creemos que el equipo en tres años propone un excesivo o poco realista número de publicaciones y presentaciones a congresos. Es relevante y se valora como un punto fuerte la publicación de un informe técnico con los resultados principales del proyecto para los profesionales que trabajen en el sector, así como la provisión del instrumento de manera abierta y gratuita. Además, el considerar dar información para las propias familias y las propias personas con DI de vídeos o folletos de prevención y divulgación, si se hace de manera correcta es asimismo otro punto fuerte. El plan de transferencia planteado es adecuado. Asimismo, es adecuado el plan de gestión propuesto asociado claramente a los resultados previstos del proyecto que se presenta. La inclusión del análisis de género en la investigación se garantiza en la mayor parte de los aspectos de la propuesta de investigación. Incluir a mujeres con DI y sus necesidades en relación a los trastornos mentales de este colectivo es claramente una

necesidad. Esta propuesta podría contribuir a una salud mental más inclusiva y social y de salud pública. La concienciación de la igualdad de género para las personas con discapacidad intelectual y, a la vez, el reconocimiento de sus particularidades y necesidades distintas como personas, ha de poder ayudar a la visibilizarían de este colectivo en nuestra sociedad.

4. Adecuación del presupuesto solicitado

El presupuesto solicitado nos parece excesivo, especialmente si tenemos en cuenta el número de investigadores que participarían en el mismo. El presupuesto solicitado para el proyecto se enfoca principalmente a la contratación de personal. Entendemos que muchos de los objetivos que se plantean, como la elaboración de la herramienta digital y su implementación, podría llevarse a cabo de otro modo, como hemos comentado con la participación de todo el personal investigador propuesto. La partida para viajes y asistencia a congresos sin embargo es muy pequeña, si tenemos en cuenta que el equipo planea la asistencia a 25 congresos en tres años. En general vemos un presupuesto muy desproporcionado, teniendo en cuenta la fuerza del equipo investigador.

PARTE 2

OTROS ASPECTOS A CONSIDERAR

A) Capacidad Formativa

El equipo de investigación tiene suficiente capacidad formativa para el contrato de una persona con una beca predoctoral. El equipo presenta una amplia lista de Tesis dirigidas y en curso, aunque no especifican el desarrollo cientifico y/o profesional de los doctores egresados. Hay poca información de la formación que recibiría esta persona y no consta la posibilidad de estancia fuera de su universidad.

B) Aspectos relacionados con zonas polares o campañas oceanográficas

No aplica

C) Condiciones específicas para la ejecución de determinados proyectos

El proyecto que se presenta tiene relación con encuestas cuantitativas en el ámbito de las ciencias sociales. Tal como se aporta, el proyecto cumple con la transferencia de microdatos. La propuesta justifica claramente los procedimientos y protocolos que usarán y disponen de todo lo necesario para su ejecución.
