# Supplementary material for: A Digital System to Assess Mental Health in People With Intellectual Disability (MENTALSED): Protocol for a Development and Pilot Study
Source: JMIR Res Protoc. 2026 Sep 30;15:e106072. doi: 10.2196/106072 (PMC13626399; doi:10.2196/106072)
Supplement: Supplementary file 2 [file resprot-v15-e106072-s002.docx]

**Scientific Technical Evaluation Report (Commission Summary)**

- **Project Reference:** PID2023-1501900A-100
- **Modality:** Type A Oriented Research
- **Area/Subarea:** Psychology
- **Principal Investigator(s):** RODRIGUEZ MEDINA, JAIRO / GONZALEZ SANGUINO, CLARA
- **Title:** MENTAL HEALTH IN PEOPLE WITH INTELLECTUAL DISABILITY. EVALUATION, DIAGNOSIS AND PREVENTION THROUGH A DIGITAL EVALUATION SYSTEM.
- **Call:** Knowledge Generation Projects 2023, Ministry of Science, Innovation and Universities / State Research Agency

**Disclaimer:** In accordance with the principles of technical discretion and independence, this scientific-technical assessment report is prepared taking into account the consensus adopted in the technical commission, after analyzing the qualitative assessments of the expert reports and the opinions of the commission experts, and in view of all the projects presented to each thematic area.

**KNOWLEDGE GENERATION PROJECTS 2023	MODALITY: TYPE A ORIENTED RESEARCH	SCIENTIFIC-TECHNICAL ASSESSMENT REPORT - TECHNICAL COMMISSION**

**PART 1**

1. **Quality and viability of the proposal**
   1.1. **Quality of the proposal:** The Commission considers that the proposal is suited to the purpose of the call, addressing a highly relevant topic such as improving the mental health of people with intellectual disabilities. To this end, it aims to develop a digital evaluation tool. The objectives are well defined and well-founded. As suggestions for improving the proposal, it is recommended to better detail the procedure for maintaining and updating the digital tool once the project is finished.

**Score 0 to 25: 22.0**

1.2. **Viability of the proposal:** The PIs have a relevant trajectory in the field of intellectual disability, which supports the viability of the proposal. The temporal planning of the project is adequate and the activities to achieve the objectives are well defined. As a recommendation, it would be advisable to detail the training plan for professionals. Also, it is recommended to provide more information on the sample size and the characteristics of the sample.

**Score 0 to 15: 12.7**

2. **Quality and trajectory of the project components:** The trajectory of both the PIs and the team is considered relevant, with the leadership capacity of the principal investigators being demonstrated. The Commission positively values the previous collaborations and joint publications among the team members, as well as the internationalization actions. The Commission has not prioritized the project to opt for a predoctoral contract for the training of doctors, given the score achieved and the number of available contracts.

**Score 0 to 30: 24.4**

3. **Expected scientific, economic and social impact of the results:** The scientific impact of the project is high, given that the creation of a digital evaluation tool can represent an advance in knowledge in the field of intellectual disability. The social impact is also considerable, by improving people's lives. The Commission considers that the transfer plan is well designed and that the results dissemination plan is adequate.

**Score 0 to 20: 17.6**

4. **Adequacy of the associated budget:** The budget is generally well justified, although the personnel expenses destined for data collection are considered excessive.

**Score 0 to 10: 6.8**

**GLOBAL EVALUATION 0 TO 100: 83.5**

### **OTHER ASPECTS TO CONSIDER**

A) Aspects related to polar zones or oceanographic campaigns *(To be completed only in applicable projects)* Not applicable.

B) Specific conditions for the execution of certain projects *(To be completed only in projects with aspects related to the conditions or implications set out in Annex IV of the call).* Not applicable.

**REPORT EXPERT 1**

1. Quality and Viability of the Proposal

1.1 Quality of the proposal

This proposal is fully suited to the modality of the call, oriented research, being a project that aims to address the design, development, and implementation of a comprehensive and digitized evaluation tool to assess mental health in people with intellectual disabilities. Likewise, it aligns with the "Health" thematic priority, working laterally on the "Inclusive society" thematic priority (due to the clear repercussions that addressing mental health in this population has for inclusion) through digital tools, technologies, and solutions for health and care (a thematic priority included in the State Plan for Scientific, Technical and Innovation Research 2021-2023).

The proposal is relevant and original, as our country does not have a tool to easily and accurately record indicators of mental health problems in people with intellectual disabilities (ID), thus contributing to diagnostic precision and, probably, to early detection and intervention. It does not present a starting hypothesis, so we cannot assess whether it is valid and original. It would be advisable (and necessary in this call) to state the expected direction of the results, supported by existing prior evidence. It develops a theoretical framework and research questions that guide the objectives, which are relevant, clearly defined, and well-founded. It is an interdisciplinary project, including professionals and experts from different fields, such as psychology, pedagogy, social work, and computer science and telecommunications.

1.2 Viability of the proposal

The previous results and contributions of the participating team and the PIs support the viability of the proposal, highlighting their extensive experience in working with people with ID, in the development and validation of evaluation instruments with this population, as well as in the proposed analyses. They have also carried out various transfer actions to society, such as the design of a comprehensive evaluation system for the Community of Madrid.

- **Methodology & Objectives:** Regarding the methodology, the objectives are presented with clarity and precision ; and the planned activities are considered adequate for them. Objective 1 is to carry out a systematic review of the scientific literature on instruments used to prevent the appearance of mental health problems in people with ID, and its methodology is adequate.
- **Sampling Concerns:** Regarding objective 2 (phase 1), there is no description of the sampling frame or an analysis of the necessary sample size, so we cannot know if the project is viable or if it will be representative. For objective 3 (phase 2), neither a sampling frame analysis nor a sample size analysis is performed. In this objective, it is mentioned that a systematic observation instrument and standardized psychometric tests will be administered, but it does not specify which specific tests will be administered, so we cannot know their pertinence. Perhaps it refers to the administration of the instruments detected and recognized as "invalid" in phase 1, but we understand that this cannot be the case (the design of an evaluation instrument must be based on the validity of other instruments, not on improving those considered invalid). Regarding objectives 4, 5, 6, and 7, there is also no description of the sampling frame or an analysis of the necessary sample size ; therefore, we cannot know if the proposal is viable in relation to the sampling frame, nor if it will be sufficient.
- **Critical Points:** They establish the critical stages along with the possible contingency plan. However, they do not consider that evaluating 600 people with ID could be a critical point, yet we assess that they may have problems with accessing the sample. Another critical point is the digitization process, which as mentioned will be in charge of an external company that will have 6 months to execute it. Perhaps this timeline is somewhat tight.

2. Quality and Trajectory of the Project Components

The previous results and contributions of the participating team and the PIs support the viability of the proposal. Thus, we can highlight an important scientific trajectory with high-impact publications in the field of ID. Some team members have been responsible for implementing the first national survey on stigma towards people with ID. Another aspect to highlight is the experience in developing and validating evaluation instruments for people with ID. Furthermore, one of the researchers is responsible for the design of a comprehensive evaluation system for people with ID in centers dependent on the Community of Madrid.

However, mutual collaborations are scarce; and some of the publications are in MDPI or Frontiers. Regarding the possibilities of internationalization, although they raise the possibility of preparing various research proposals derived from the project for the ERASMUS+ call, they do not have any international member to facilitate this initiative.

3. Expected Scientific, Economic, and Social Impact of the Results

The proposal has a high scientific impact, as it explores the mental health of people with ID, a highly novel field of study. At a social level, it involves the development of a tool that could have a considerable impact in the field of disability, since it will be computerized and offered free of charge to entities and professionals working with this population. Additionally, the project will contribute to improving diagnostic tools for mental health disorders, which could lead to early detection—and consequent intervention—and this in turn could result in a reduction of healthcare and medication costs.

- **Dissemination and Transfer:** It has a results dissemination plan, focused on a technical report, accessible dissemination materials, and publications in dissemination media (it does not indicate which ones). The transfer plan focuses on disseminating results at congresses, workshops, and scientific publications. We believe, upon reading the proposal, that this plan overlaps with the scientific dissemination proposal. In the latter, they mention 15 articles, but they do not describe the milestones or specific themes each of these will cover, nor the proposed journals. We estimate that this project may yield publications of interest and impact, but 15 articles are probably too many for its objectives.
- **Social Reach:** We value the interest shown by the PIs in ensuring the project's results reach patients, families, and professionals ; highlighting the inclusion of scientific-technical support activities for third-sector entities that require expert knowledge to use the digital evaluation system or to plan applied studies, and the interest in developing training material aimed at interested researchers, professionals, families, and people with ID.
- **Data Management & Inclusion:** It has an adequate management plan for the project's results, highlighting that the results will be findable, accessible, interoperable, and reusable in the context of scientific research (FAIR principles). The proposal contemplates the inclusion of the gender perspective in its objectives, activities, and methodology. We recommend supporting this inclusion with the theoretical framework, as there is extensive literature evidencing gender inequalities in the fields of mental health and disability. The field of disability is intrinsic to the proposal itself.

4. Adequacy of the Requested Budget

The items are sufficiently detailed and justified. It requests an allocation of 6000 Euros for the purchase of Polar H10 heart rate variability data collection bands (approx. 3000 euros), for IT equipment (2000 euros), and for other associated expenses (1000 euros), but it does not detail what expenses are included in this allocation. An allocation for open-access publications is also included. In this regard, the current call recommends using free repositories, such as those available at most universities or Digital. CSIC ; and not specific budget allocations for these expenses.

**OTHER ASPECTS TO CONSIDER**

A) Training Capacity The training that will be provided from the doctoral program in Transdisciplinary Research in Education at the University of Valladolid is detailed, which includes training activities on research methodology, attendance at congresses, and the publication of—at least—one scientific article. They list 16 doctoral theses defended in recent years, two of them on the topic of disability; the rest are specifically focused on executive functioning and neuroeducation. They do not describe the scientific and/or professional development of the doctors who have graduated from the research team.

B) Aspects related to polar zones or oceanographic campaigns NA

C) Specific conditions for the execution of certain projects It describes the specific considerations that will be taken into account when working with human beings.

**REPORT EXPERT 2**

## 1. Quality and Viability of the Proposal

### 1.1 Quality of the proposal

The proposal is suited to the characteristics and purpose of the call, focusing on improving the mental health of people with intellectual disabilities (PWID) through the development of a digital evaluation and diagnostic tool.

- Alignment and Relevance: This proposal is aligned with the thematic priority of Health and Inclusive Society, as detailed in the State Plan for Scientific, Technical and Innovation Research 2021-2023. Specifically, it focuses on digital tools and solutions for health and care, as well as promoting an inclusive society by addressing the barriers faced by PWID. The proposal is highly relevant due to the significant unmet need for diagnostic tools adapted for PWID.
- Hypothesis and Objectives: The starting hypothesis is valid and original, pointing out that PWID may have comorbid psychopathologies more frequently than the general population, but their diagnosis is complicated due to the lack of adapted tools. The proposal to develop a digital tool that combines the evaluation of behavioral problems, mental health, and emotional well-being is innovative and directly addresses this need. The general objectives are ambitious, relevant, and clearly defined. They are also realistic given the work plan and the proposed interdisciplinary methodology, which are based on proven methodologies and the previous experience of the research team.
- Strengths: The proposal adequately justifies its multidisciplinary approach. It involves professionals from different disciplines, allowing the problem to be addressed from different perspectives. Collaboration with various universities and entities, both nationally and internationally, strengthens the viability and potential impact of the project.
- Weaknesses: * The project's success depends heavily on the development and adoption of the digital tool. Any delay or failure in technological implementation could compromise the achievement of the objectives.
  - The interdisciplinary methodology may present problems in coordinating and integrating different disciplines and approaches, potentially resulting in execution delays.
  - Given the variability among PWID, the results and tools developed may not be applicable to all subpopulations within this group; personalizing and adapting the tool entails considerable difficulty.
  - The proposal lacks in-depth details regarding plans for the sustainability and scalability of the tool once the project ends (e.g., maintenance, updates, and guaranteeing long-term adoption).
  - The proposal relies on obtaining information during the project to define the tool, rather than basing it on prior literature and existing developments. A clearer definition based on previous research would be advisable to ensure the tool is built on a proven foundation.

### 1.2 Viability of the proposal

The members of the research team, and in particular the two PIs, have a relevant trajectory in the field of clinical psychology and mental health, especially in intellectual disability contexts. Both PIs have significant publications and experience in related projects, which supports the proposal's viability.

- Timeline and Methodology: The specific objectives are clearly defined and align with the project's duration and the team's composition. The objectives are logically organized chronologically, allowing a natural progression from the systematic literature review to the development and validation of the digital tool. The temporal planning seems adequate. The described activities are well structured, and the proposal demonstrates an understanding of methodological difficulties while offering solutions.
- Risks and Weaknesses: * Directing an interdisciplinary team involves difficulties that make a more developed coordination plan advisable.
  - Collecting high-quality empirical data from a vulnerable population like PWID can present ethical and logistical problems.
  - Dependence on technological development represents a risk; unforeseen technical issues could compromise the objectives. It would be beneficial to include more detailed technical specifications from the start, based on prior literature.
- Contingency Plan: The project identifies critical stages that could affect viability (e.g., technological development and empirical data collection). The proposed contingency plan is generally adequate and contemplates reasonable alternatives to mitigate some risks (schedule adjustments, activity restructuring, and additional resources). However, for other risks, the planned measures do not seem sufficient, relying solely on their low probability of occurrence.

## 2. Quality and Trajectory of the Project Components

This is a large team with a solid trajectory.

- Leadership: PI-1 has published numerous articles and participated in several competitive research projects; his experience in interdisciplinary projects and publication record validate him as a suitable leader. PI-2 has extensive experience researching mental health and stigma, numerous publications, and recognized research awards; her background also makes her well-suited to co-lead.
- Team Composition: The rest of the team includes members specialized in psychology, pedagogy, social practice, computer science, and telecommunications. This diversity of knowledge is appropriate for the project's development.
- Collaboration: There is evidence of prior collaboration among team members (co-authorships and joint research projects), and this cohesion strengthens the project's viability. They also maintain active collaborations with international groups and entities in Europe and the Americas.

## 3. Expected Scientific, Economic, and Social Impact of the Results

The project can have a relevant scientific-technical impact. It addresses a clear need in the evaluation and diagnosis of mental health in PWID, an area that has been insufficiently researched.

- Social and Economic Impact: Developing an innovative digital tool can establish new standards in this field. The social impact is potentially significant: it can improve the quality of life of PWID by facilitating more precise and earlier diagnoses, allowing for more effective interventions. This benefits families, reduces stigma, and promotes social inclusion. Economically, the tool can save resources by improving evaluation efficiency and reducing the need for intensive, costly long-term treatments.
- Transfer and Dissemination Plan: The transfer and valorization plan is well-designed. It includes collaboration with third-sector professionals and entities working directly with PWID, ensuring real-world relevance. End-user participation (direct care professionals and families) in the tool's design and validation strengthens its applicability. The dissemination plan appropriately targets both the scientific community and the general public.
- Weaknesses: The scientific production forecasts indicated in the proposal seem unrealistic. Expecting 20 indexed journal publications, 10 in other journals, 2 books, 10 book chapters, 20 international congress presentations, and 20 national congress presentations in a single project is excessively ambitious and does not align with typical production rates for projects of this size or the team's historical output. It would be wiser to adjust these figures to ensure publication quality.
- Data and Inclusion: The proposal includes an adequate research data management plan and properly addresses the gender dimension (considering the inclusion of women and the elderly within the PWID population, with specific methodologies for these subgroups).

## 4. Adequacy of the Requested Budget

- Personnel: The hiring of personnel to support data collection is requested. Given the extensive number of members on the research and work teams, the need for this external support is not entirely clear.
- Equipment: It is necessary to specify the IT equipment included in the inventoriable section.
- Travel/Publications: The budget for participating in congresses and publishing seems insufficient, given the very high number of congresses they wish to attend and the vast number of publications planned.

### **OTHER ASPECTS TO CONSIDER**

A) Training Capacity The planned training program is adequate, although it would be advisable to specify the specific training activities to a greater extent, beyond those programmed within the framework of the doctoral program. The PIs do not yet have extensive experience in directing theses, but the research team as a whole does, with a long list of doctoral students who have passed through the group. It would also be advisable to include the completion of stays at other centers, both national and international, in the training program.

B) Aspects related to polar zones or oceanographic campaigns.

C) Specific conditions for the execution of certain projects The proposal adequately justifies the procedures and protocols it plans to apply.

**REPORT EXPERT 3**

## 1. Quality and Viability of the Proposal

### 1.1 Quality of the proposal

- Alignment and Relevance: The project is suited to the call and its modalities (Strategic Action 1 - Health). It also aligns with the thematic priority (Health - Digital tools, technologies, and solutions for health and care). Both points are clearly explained at the beginning of the Memory. It is also linked to Strategic Action 2 (Culture, creativity, and Inclusive Society).
- Hypothesis and Objectives: The project is relevant and innovative. The hypothesis is well-structured and original in its approach. The objectives are clear, realistic, and very ambitious, partly due to the number of participants required to achieve the various proposed goals.
- Methodology: The project is clearly multidisciplinary, and the inclusion of different areas of knowledge (with their respective specialists within the research team) is clearly justified in the methodology and relevant for achieving the proposed objectives.

### 1.2 Viability of the proposal

- Team Experience: The project is clearly viable given the trajectory of the research team, which demonstrates their experience in the project's topic. This trajectory is supported by high-impact publications and funded research projects.
- Planning and Competence: The specific objectives are well explained and consistent with the proposed timeline for developing the project. The research team is competent and adequate for achieving the objectives, and there is gender balance.
- Contingency Plan: The contingency plan is correct, although it does not address the possibility of low participation from the involved groups of individuals (patients, professionals).

## 2. Quality and Trajectory of the Project Components

- PI-1: IP-1 has a good CV demonstrating experience in the project's topic, corroborated by a respectable number of publications and funded research projects, some at the European level. Their training capacity is supported by the direction of 4 Doctoral Theses. There are no records of transfer to industry or society. Apparently, IP-1 should have the capacity to lead this project.
- PI-2: IP-2 also has a good CV, not as extensive as IP-1 (logical due to age difference), but which complements that of IP-1 in the field of Psychology. They have participated in international collaborative teaching activities (PAHO). Their training capacity includes the co-direction of a Doctoral Thesis and Master's Theses, as well as teaching at the Complutense University of Madrid and the University of Valladolid.
- Team Composition: The contributions of the research team members, as a whole, are important, and their trajectories guarantee the achievement of the proposed objectives in the project. Two researchers (MM and MJIM) stand out with excellent CVs in areas of knowledge relevant to the project.
- International Scope: The research team's international collaborations are limited.

## 3. Expected Scientific, Economic, and Social Impact of the Results

- Impact Potential: The scientific and economic impact can be relevant, but the social impact will be even more so if the project's objectives are met, which seems probable when assessing the proposal and the research team.
- Transfer and Dissemination: The results transfer plan is excellent, realistic, and very well explained in the memory. End users participate in several methodological areas. The dissemination/outreach plan is correct and realistic, including the management of research data in relation to the achieved objectives, especially its application to the target audience in society.
- Gender Equality: In the gender area, the proposal includes specific mentions of equality for both the participants (research team) and the targets of the research (people with disabilities).

## 4. Adequacy of the Requested Budget

The budget is suited to the proposed objectives and is detailed across the different types of expenses.

**OTHER ASPECTS TO CONSIDER**

A) Training Capacity N/A

B) Aspects related to polar zones or oceanographic campaigns N/A

C) Specific conditions for the execution of certain projects N/A

**REPORT EXPERT 4**

1. Quality and Viability of the Proposal

1.1 Quality of the proposal

Dear researchers, thank you for giving me the opportunity to review your interesting project. The presented research project is perfectly suited to the characteristics and purpose of the selected modality (Health and Inclusive Society). People with intellectual disabilities (ID) have a higher risk of presenting major mental health disorders than the general population, a point that is clearly argued and justified. Therefore, the need to evaluate and have assessment instruments available as soon as possible is a clear necessity that must be addressed to improve the health of people with intellectual disabilities, their quality of life, and ultimately, their inclusion in our society. These instruments must be valid, reliable, and functional in their application, especially for the end users. Helping the various professionals who attend to people with ID to diagnose Mental Disorders remains a highly necessary challenge.

The purpose of having an automated recording tool is very novel and, at the same time, useful and tailored to the contexts in which it will be used (day centers, special employment centers, and residences). The general objective is clear, as are the specific objectives. The project is very well delimited into phases, and the methodology used to achieve each specific objective is described. Different methodological approaches are proposed to achieve the various objectives, complementing one another to reach the overall goal of the proposal. The objectives are realistic and well-adjusted.

1.2 Viability of the proposal

Clearly, the previous contributions of the participating team guarantee the viability of the proposal. As previously mentioned, each of the six proposed objectives is very clear and supported by methodologies appropriate for the intended purposes. The timeline is adequate, and the contingency plan is appropriate. Particularly important is the collaboration with participating entities from the outset. It seems that some third-sector entities have been contacted, although I clearly believe that additional ones should be contacted in case of dropout or non-participation. It should be expanded upon what the training plan for professionals would consist of and how the learning of the content and its practical application would be guaranteed.

2. Quality and Trajectory of the Project Components

The research team is composed of various researchers from the University of Valladolid belonging to a Recognized Research Group. The research team's previous work with *Plena Inclusión*—a clearly recognized entity in Spain that groups together various professionals and families of people with intellectual disabilities—is acknowledged. The report they present on a study carried out in 2021 is complete and supports the team's capacity for collaborative work with third-sector entities.

The team's Principal Investigator has a recognized, solid academic and research profile with extensive publications on the topic presented. The Second Principal Investigator does not have as broad or extensive a research profile, although it aligns with the topic demanded in their research. The team, in general, has published numerous articles both nationally and internationally, as well as book chapters and various presentations at both national and international congresses. We have no doubt of their value to lead and carry out the project with sufficient guarantees. The rest of the team members also share common interests and hold strong CVs. Noteworthy is the collaboration with other universities (Complutense University of Madrid, University of Zaragoza, Ramón Llull University, National University of Mar del Plata), which can clearly enrich the project. The continued collaboration with *Plena Inclusión* through service-learning initiatives prior to this research project is mentioned as a merit.

3. Expected Scientific, Economic, and Social Impact of the Results

The presented research project offers significant benefits in the field of mental health for people with intellectual disabilities. Knowing the prevalence and types of mental disorders, how to detect them in time, and how to address them is, in itself, an important advance with a clear social and community impact that would allow for the development of new action policies for this collective. We believe that the project has a greater impact at the national level than internationally, although the development of a digital tool like the one proposed in the project can serve as a starting point or comparison with other countries. On the other hand, the social impact for the inclusion of people with intellectual disabilities is clear, as it will allow for deeper understanding of this collective and their mental health disorder issues associated with this condition, which must be treated appropriately and efficiently. Economically, it is not as clear that it can represent a gain unless accompanied by sufficient changes at the level of general public health.

Regarding the plan for scientific communication and internationalization of the results, we believe that the team proposes an excessive or unrealistic number of publications and congress presentations over three years. Publishing a technical report with the project's main results for professionals working in the sector, as well as providing the instrument openly and for free, is highly relevant and valued as a strong point. Furthermore, considering providing information for the families themselves and people with ID through prevention and outreach videos or brochures, if done correctly, is also another strong point.

The proposed transfer plan is adequate, as is the proposed management plan, which is clearly associated with the expected results of the presented project. The inclusion of gender analysis in the research is guaranteed in most aspects of the research proposal. Including women with ID and their needs in relation to the mental disorders of this collective is clearly a necessity; this proposal could contribute to a more inclusive and social public mental health approach. Raising awareness of gender equality for people with intellectual disabilities and, at the same time, recognizing their particularities and distinct needs as individuals, must be able to help make this collective visible in our society.

4. Adequacy of the Requested Budget

The requested budget seems excessive to us, especially when taking into account the number of researchers who would participate in it. The budget requested for the project is focused mainly on hiring personnel. We understand that many of the proposed objectives, such as the development of the digital tool and its implementation, could be carried out in another way, as we have commented, with the participation of all the proposed research staff. The allocation for travel and congress attendance, however, is very small, considering that the team plans to attend 25 congresses in three years. In general, we see a very disproportionate budget, considering the strength of the research team.

**OTHER ASPECTS TO CONSIDER**

A) Training Capacity The research team has sufficient training capacity to host a predoctoral contract. The team presents an extensive list of directed and ongoing theses, although they do not specify the scientific and/or professional development of the graduated doctors. There is little information regarding the training this person would receive, and there is no mention of the possibility of a stay outside their university.

B) Aspects related to polar zones or oceanographic campaigns Not applicable

C) Specific conditions for the execution of certain projects The presented project is related to quantitative surveys in the field of social sciences. As provided, the project complies with the transfer of microdata.
